# Acute toxicity of normofractionated intensity modulated radiotherapy with simultaneous integrated boost compared to three-dimensional conformal radiotherapy with sequential boost in the adjuvant treatment of breast cancer

**DOI:** 10.1186/s13014-020-01652-x

**Published:** 2020-10-13

**Authors:** David Krug, Christine Köder, Matthias F. Häfner, Nathalie Arians, Semi B. Harrabi, Stefan A. Koerber, Tobias Forster, Ingmar Schlampp, Christof Sohn, Joerg Heil, Holger Hof, Juliane Hörner-Rieber, Jürgen Debus

**Affiliations:** 1 Department of Radiation Oncology, Heidelberg University Hospital, Heidelberg, Germany; 2 Heidelberg Institute of Radiation Oncology (HIRO), National Center for Radiation Research in Oncology (NCRO), Heidelberg, Germany; 3 Department of Radiation Oncology, University Hospital Schleswig-Holstein, Campus Kiel, Arnold-Heller-Str. 3, 24105 Kiel, Germany; 4 Department of Gynecology and Obstetrics, Heidelberg University Hospital, Heidelberg, Germany; 5 Strahlentherapie Rhein-Pfalz, Neustadt, Germany; 6 German Cancer Consortium (DKTK), Partner site Heidelberg, Heidelberg, Germany

**Keywords:** Boost irradiation, Breast-conserving surgery, Radiation dermatitis, Breast pain

## Abstract

**Background:**

Intensity-modulated radiotherapy (IMRT) improves dose homogeneity and late toxicity compared to simple tangential techniques in adjuvant whole-breast radiotherapy for patients with breast cancer. Simultaneous-integrated boost (SIB) radiotherapy shortens the overall treatment time and improves dose homogeneity. However, prospective randomized trials regarding IMRT with SIB for adjuvant radiotherapy in breast cancer are lacking.

**Methods:**

The IMRT-MC2 (MINT) trial is a phase III prospective randomized controlled trial comparing IMRT with SIB (Arm A: whole breast 28 × 1.8 Gy, Boost 28 × 2.3 Gy) to 3D-conformal radiotherapy with a sequential boost (Arm B: whole breast 28 × 1.8 Gy, boost 8 × 2 Gy) in patients with breast cancer after BCS. Indication for boost radiotherapy was defined as age < 70 years or age > 70 years with presence of additional risk factors. This is a retrospective analysis of acute toxicity at one of two trial sites.

**Results:**

Five hundred two patients were randomized, of which 446 patients were eligible for this analysis. There was no statistically significant difference in terms of any grade radiation dermatitis between the two treatment arms at the end of treatment (*p* = 0.26). However, radiation dermatitis grade 2/3 (29.1% vs. 20.1 and 3.5% vs. 2.3%) occurred significantly more often in Arm A (*p* = 0.02). Breast/chest wall pain at the first follow-up visit was significantly more common in patients treated on Arm B (p = 0.02).

**Conclusions:**

Treatment on both arms was well tolerated, however there were some differences regarding radiodermatitis and breast pain. Further analyses are ongoing.

**Trial registration:**

clinicaltrials.gov, NCT01322854, registered 24th March 2011.

## Background

Adjuvant whole-breast radiotherapy is standard after breast-conserving surgery (BCS) for invasive breast cancer. The 2011 meta-analysis by the Early Breast Cancer Trialists’ Collaborative Group showed a 15.7% reduction in any breast cancer recurrence 10 years after treatment, resulting in an 3.8% improvement in breast cancer-specific survival at 15 years [1]. Local control can be further improved by the addition of a boost to the tumor bed. The 20 year-results of the EORTC boost-trial showed an absolute 4.4%-reduction of local recurrence (hazard ratio 0.65) [2]. In this trial, 16 Gy in 8 fractions were delivered to the tumor bed using electrons or photons or 10 Gy via pulsed dose rate-brachytherapy. In the Lyon-trial, 10 Gy in 4 fractions were given with electrons, resulting in an absolute 0.9%-reduction of local recurrence at 5 years. In the trials establishing hypofractionated whole-breast radiotherapy, boost irradiation was also administered sequentially, typically with a single dose of 2 Gy and a total dose of 10–16 Gy [3–5]. Nowadays, modern irradiation techniques allow to deliver the boost simultaneously to whole breast radiotherapy by delivering a slightly higher dose (typically 0.3–0.5 Gy) to the tumor bed at each fraction. This technique is called simultaneous integrated boost (SIB) and has been used in a variety of diseases.

Planning studies have suggested improved dose homogeneity as well as advantages in terms of a reduction in mean biological breast and lung doses for SIB as compared to sequential boost irradiation in patients with breast cancer [6–9]. The role of intensity-modulated radiotherapy (IMRT) for patients with breast cancer has been studied in three randomized controlled trials. However, these trials used forward planned IMRT with field in field-tangents in the experimental arms compared to two-dimensional radiotherapy in the standard arms [10–12]. All three trials demonstrated improvements in dose homogeneity [10, 11, 13]. A reduction in acute toxicity was only demonstrated in the trial by Pignol et al. and a reduction in late toxicity in the trials by Donovan et al. and in the Cambridge IMRT-trial [14].

The IMRT-MC2 (MINT) trial is a randomized controlled phase III-trial addressing the role of IMRT-SIB in the adjuvant treatment of patients with breast cancer after BCS. This publication analyzes the acute toxicity of IMRT-SIB compared to three dimensional-conformal radiotherapy (3D-CRT) with a sequential boost (seqB) [15].

## Methods

The IMRT-MC2 (MINT)-trial is a randomized phase III-trial (NCT01322854). The study protocol has been published previously [15]. Patients with breast cancer treated with breast-conserving surgery and an indication for boost radiotherapy (age < 70 years or age ≥ 70 years with risk factors for local recurrence: tumor size > 2 cm, multifocality, lymphangiosis, extensive intraductal component, surgical margin ≤3 mm) were included. Exclusion criteria were Karnofsky performance index ≤70%, distant metastases, diagnosis of a previous malignant tumor ≤5 years prior to enrollment, previous chest radiotherapy, pregnancy and mental disorders with impaired comprehension of relevant aspects of this clinical trial.

Patients randomized to the experimental arm (Arm A) received inverse-planned IMRT of 50.4 Gy in 28 fractions to the whole breast with a SIB of 64.4 Gy in 28 fractions (single dose 2.3 Gy) to the tumor bed. Patients randomized to the standard arm (Arm B) were treated with 3D-CRT of 50.4 Gy in 28 fractions to the whole breast with a seqB of 16 Gy in 8 fractions to the tumor bed. Most patients in Arm B received treatment with field-in-field tangents. Regional nodal irradiation was permitted in both arms. Randomization was stratified by treatment center, tumor size (T1 vs. ≥T2), cosmetic outcome prior to initiation of radiotherapy (excellent/good vs. fair/poor) and use of chemotherapy and/or targeted therapy. The co-primary endpoints are local control after two and 5 years and cosmetic outcome after two years as assessed by the breast retraction assessment [16, 17]. Secondary endpoints include toxicity using the LENT-SOMA classification, disease-free survival, overall survival, quality of life, cosmetic outcome using the Harvard-scale [18]. Patients were assessed prior to radiotherapy, 6–8 weeks, 2 years and 5 years after the end of radiotherapy.

This is a retrospective analysis of all patients at one of two recruiting centers (Heidelberg University Hospital). Patient charts, study documentation as well as treatment and follow up reports during treatment and at the first follow up visit 6–8 weeks after treatment were assessed by two investigators (DK and CK). Radiation dermatitis, pneumonitis and breast/chest wall pain were evaluated and categorized according to the CTCAE 4.03 criteria, which were used for documentation in clinical routine during the conduct of the trial. Furthermore, lymphedema was grouped according to a local score: 0 = no symptoms or treatment, 1 = symptoms of lymphedema but no specific treatment, 2 = compression therapy by means of manual or physical decompression therapy.

The IMRT-MC2 (MINT)-trial was approved by the local ethics committee at Heidelberg University and by the federal regulatory agency for radiation protection (Bundesamt für Strahlenschutz). The trial was registered at clinicalrials.gov (NCT01322854).

### Statistical analysis

Data were managed using Microsoft Excel for Mac version 16.32 (Microsoft Corporation, Redmond, USA). Statistical analyses were conducted using GraphPad Prism 5 (GraphPad Software, San Diego, USA). Baseline characteristics were compared using the Mann-Whitney-U-test and chi^2^-test. Wilcoxon matched-pairs signed rank-test was used for comparison of ordinal data. For analysis of nominal binomial variables, chi^2^-test was used. A *p*-value ≤0.05 was considered statistically significant.

## Results

**Fig. 1:**
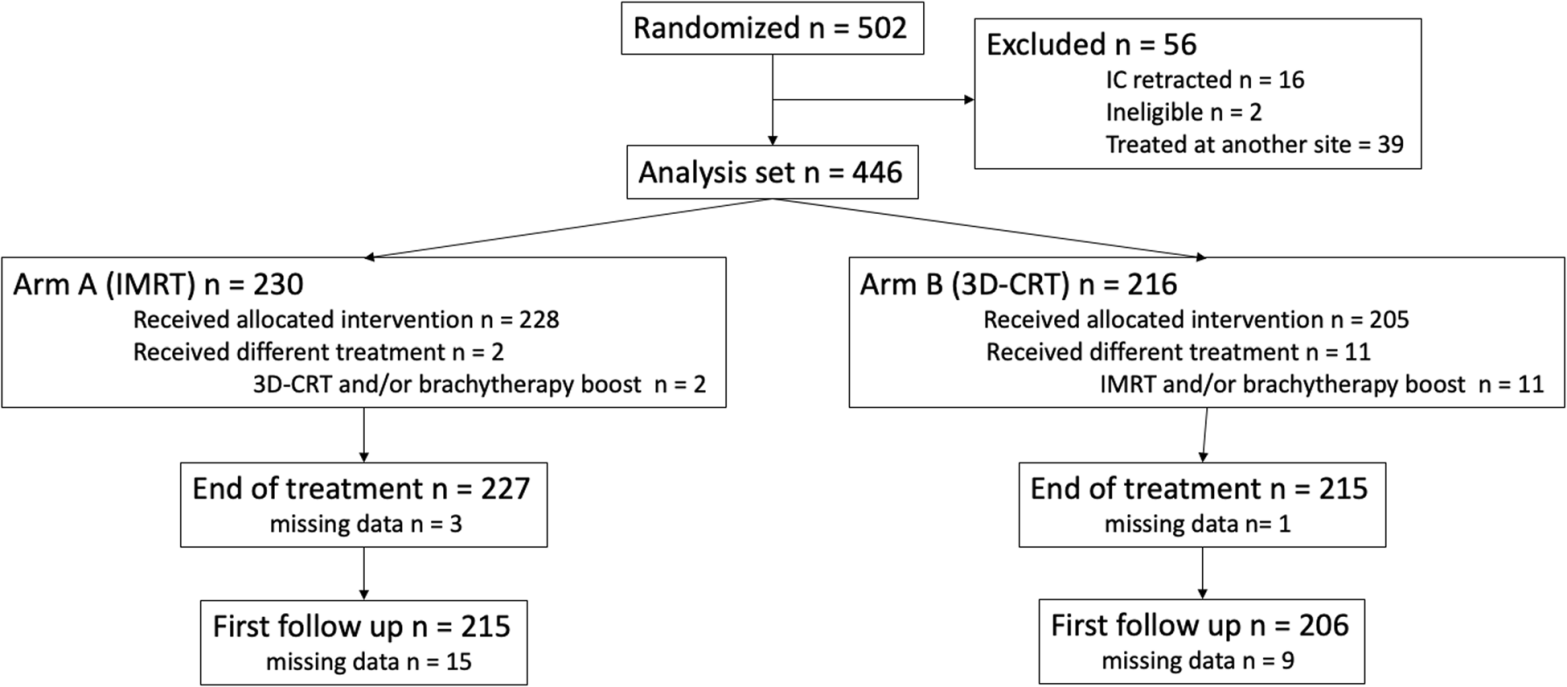
CONSORT-diagram. IC = informed consent; IMRT = intensity-modulated radiotherapy; SIB = simultaneous integrated boost; 3D-CRT = 3D-conformal radiotherapy; seqB = sequential boost

From 2011 to 2015, 502 patients were randomized. Figure 1 shows the CONSORT-diagram for this retrospective analysis. After exclusion of patients recruited at the other trial site, ineligible patients and patients who withdrew from the trial, 446 patients remained for the analysis. Most patients on Arm A received tomotherapy (78.7%), the remaining patients had step-and-shoot IMRT or volumetric modulated arc therapy. 19% of patients in Arm B received a reduced boost dose of 5 × 2 Gy, which was not considered a major protocol violation according to prespecified criteria.

Table 1 shows the patient characteristics. Baseline parameters were well balanced between the two groups. Most patients had pT1–2 tumors and had no involved lymph nodes. 43.5% of patients received any sort of chemotherapy, of which 36.6% had neoadjuvant chemotherapy. 14.8% of patients received regional nodal irradiation.

**Table 1:** Baseline characteristics

	All patients (446)	Arm A (230) IMRT/SIB	Arm B (216) 3D-CRT/seqB	
Age (median)	55.7	56.0	55.3	*p* = 0.44
Tumor stage				
pT1–2	368 (82.5%)	189 (82.1%)	179 (82.9%)	*p* = 0.29
pT3–4	7 (1.6%)	5 (2.2%)	2 (0.9%)	
cT1–2	69 (15.5%)	34 (14.8%)	35 (16.2%)	*p* = 0.16
cT3–4	2 (0.4%)	2 (0.4%)	0	
Nodal stage				
pN0	295 (66.1%)	155 (67.4%)	140 (64.8%)	*p* = 0.55
pN1–3	80 (33.9%)	39 (32.6%)	41 (35.2%)	
cN0	50 (11.2%)	23 (10.0%)	27 (12.5%)	*p* = 0.22
cN+	21 (4.7%)	13 (5.7%)	8 (3.7%)	
Regional nodal irradiation	66 (14.8%)	28 (12.2%)	38 (17.6%)	*p* = 0.11
Chemotherapy				*p* = 0.99
adjuvant	194 (43.5%)	100 (43.5%)	94 (43.5%)	
neoadjuvant	71 (36.6%)	36 (36.0%)	35 (37.2%)	

*IMRT* intensity-modulated radiotherapy, *SIB* simultaneous integrated boost, *seqB* sequential boost

**Fig. 2:**
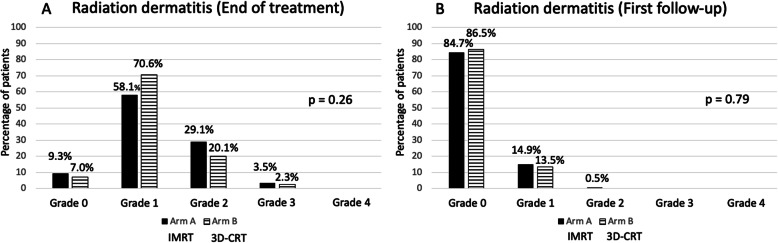
Radiation dermatitis at end of treatment (**a**) and first follow-up (**b**; 6–8 weeks after treatment)

The prevalence of radiation dermatitis at the end of treatment according to CTCAE-grading is shown in Fig. 2. There was no statistically significant difference in terms of radiation dermatitis between the two treatment arms (*p* = 0.26). However, radiation dermatitis grade ≥ 2 (29.1% vs. 20.1 and 3.5% vs. 2.3%) occurred significantly more often in Arm A (*p* = 0.02). This was also true when excluding patients who received a boost dose of 10 Gy in Arm B (*p* = 0.01).

**Fig. 3:**
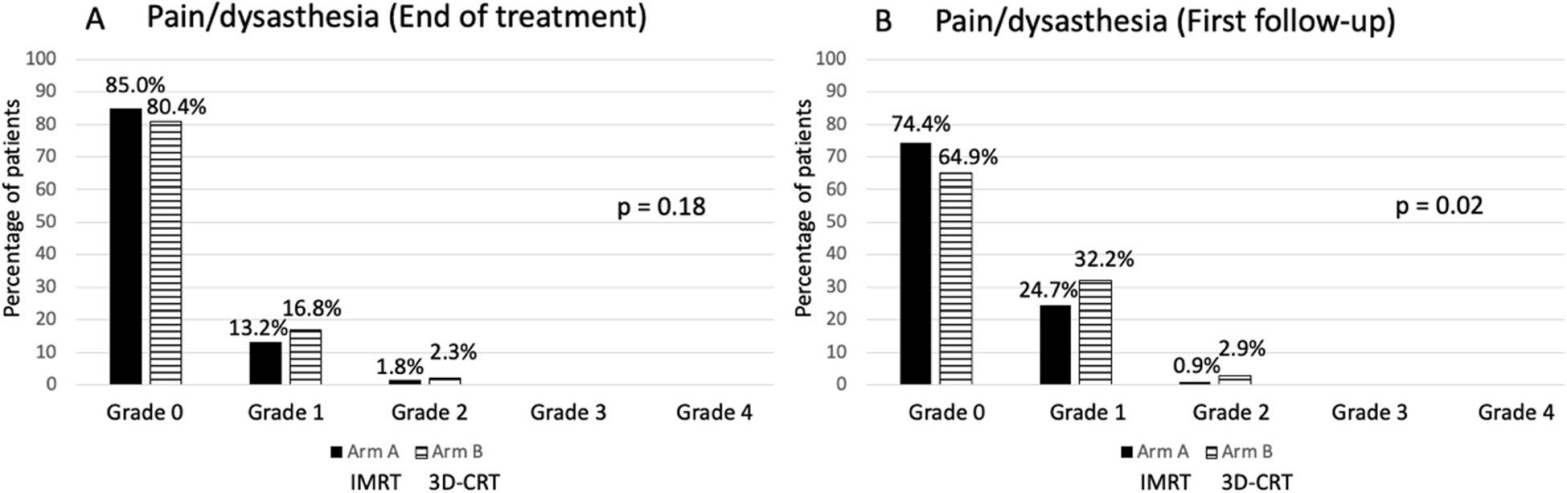
Pain/dysesthesia at end of treatment (**a**) and first follow-up (**b**; 6–8 weeks after treatment)

As shown in Fig. 3, at the first follow-up visit, 14.9% (Arm A) and 13.5% (Arm B) had residual radiation dermatitis grade 1 (*p* = 0.79). One patient in Arm A had residual radiation dermatitis grade 2. Breast/chest wall pain at the end of treatment was numerically higher in patients treated on Arm B (*p* = 0.18; Fig. 3a). The prevalence of pain grade 1 and grade 2 was 13.2 and 1.8% on Arm A and 16.8 and 2.3% on Arm B. The prevalence of breast/chest wall pain was higher at the first follow-up visit for both treatment arms (Fig. 3b). The prevalence of breast/chest wall pain at the first follow-up visit was significantly higher for patients on Arm B as compared to Arm A (*p* = 0.02). The prevalence of breast/chest wall pain for Arm A and Arm B at the first follow-up was 24.7% vs. 32.2% for grade 1 and 0.9% vs. 2.9% for grade 2-events. Clinical or radiologic signs of pneumonitis (grade 1) did only occur at the end of treatment and were seen in 1.8% of patients on Arm A and 0% of patients in Arm B (*p* = ns). Lymphedema grade 1–2 was present in 5.3% vs. 5.6% of patients at the end of treatment and in 7.4% vs. 9.1% of patients at the first follow-up visit.

Two major treatment interruptions (more than 4 days) occurred due to decompensated liver cirrhosis, and mastitis in 1 patient each. There were no grade 3–5-events at the first follow-up visit.

## Discussion

This retrospective analysis of a prospective randomized controlled phase III-trial shows similar acute toxicities in patients treated with 3D-CRT-seqB and IMRT-SIB. There was a significantly higher incidence of thoracic wall/breast pain in patients with 3D-CRT-seqB at the first follow-up visit and higher incidence of radiation dermatitis grade 2–3 in patients treated with IMRT-SIB at the end of treatment. All other outcome parameters showed similar rates of acute toxicity.

Three randomized controlled trials studied the role of IMRT in breast cancer [10–12, 14]. However, they used another definition of IMRT since both trials employed a forward-planned tangential field-in-field technique in the experimental arms, which was compared to two dimensional (2D) tangential radiotherapy. The trial by Pignol et al. showed a significant reduction in acute skin toxicity in the IMRT-arm, especially in the occurrence of moist desquamation [11]. There was no difference in long-term toxicity and morbidity between the two treatment arms, although there was a significant correlation between acute moist desquamation and late subcutaneous fibrosis and teleangiectasia [12]. In contrast, the trial by Donovan et al. demonstrated a significant reduction in changes in breast appearance and palpable induration in the IMRT-arm [10]. This reduction in chronic toxicity is supported by the results of the Cambridge IMRT-trial, which showed a significantly lower risk of teleangiectasia and suboptimal overall cosmesis in patients randomly assigned to forward-planned IMRT as compared to 2D-planned radiotherapy [14]. Thus, dose homogeneity might affect acute and chronic toxicity [19].

Shortening the overall treatment time by integrating the boost irradiation into a normofractionated whole-breast treatment (NF) course, resulting in a simultaneous integrated boost, has been studied in several single-arm studies [20–22]. A large single-center analysis from the Netherlands showed favorable outcomes regarding local control [21] as well as morbidity and cosmesis [20] with a regimen of 50.4 Gy in 28 fractions to the whole breast and 64.4 Gy (single doses 2.3 Gy) or 67.2 Gy (single dose 2.4 Gy, in case of focally positive surgical margins) to the tumor bed using 3D-CRT. While results were favorable for all outcome parameters, acute toxicity was not reported. McDonald et al. published results for multi-angle IMRT using several different 25/28-fraction regimens with a median follow-up of 33 months [22]. Acute grade 2-radiodermatis was experienced by 43% of patients, which was considerably higher than in our study. Pasquier et al. conducted a prospective trial of NF-SIB with tomotherapy in 119 breast cancer patients and found comparable rates of acute radiodermatitis with grade 1, 2 and 3-toxicity occuring in 66.1, 22.3 and 2.1% of patients, respectively [23]. Meng et al. [24] recently reported their experience of 467 treated with NF-SIB (45 Gy to the whole breast and 60 Gy to the tumor bed in 25 fractions delivered with IMRT, no further details were provided regarding treatment planning). Grade 2-dermatitis occurred in 23.5% of patients, no grade 3-dermatitis was recorded. Interestingly, they found radiographic features suggestive of pneumonitis in 25.3% of patients at a routine CT-scan 6 months after treatment. No patient was symptomatic. There is few data for comparison, however Vasiljevic et al. [25] conducted a prospective study of 100 patients with follow-up CT-scans at 13 and 24 weeks after RT. They identified 13 patients with features suggestive of pneumonitis, all of which had mild respiratory symptoms. Our incidence of pneumonitis was remarkably lower (1.8%), however no routine chest imaging was conducted and pneumonitis often occurs later than 6–8 weeks after treatment.

A retrospective comparative analysis for seqB vs. SIB including 126 patients was published in 2015 [26]. The IMRT-SIB patients were treated with helical tomotherapy and received 50.4 Gy to the whole breast and 60.2 Gy (single dose 2.15 Gy) to the tumor bed in 28 fractions. There was a significantly higher incidence of grade 2 radiodermatitis in the 3D-CRT-seqB-group. Local control, disease-free survival and overall survival at 3 years were similar between the two groups. There are two small randomized controlled trials studying hypofractionated whole-breast radiotherapy (HF) and comparing SIB to seqB [27, 28]. Both trials showed a reduction of acute skin toxicity by the incorporation of SIB. However, the overall incidence of radiation dermatitis is significantly lower with hypofractionated radiotherapy [29] and one trial used prone radiotherapy [27], which might impair the cross-trial comparison. Two retrospective studies of HF-SIB and NF-SIB have been published recently. Fiorentino et al. [30] studied 80 patients treated either with IMRT and NF-SIB (50/60 Gy in 25 fractions) or VMAT and HF-SIB (40.5 Gy and 48 Gy in 15 fractions). Acute skin toxicity was significantly lower for HF-SIB with 2.5% compared to 25% with NF-SIB. For NF-SIB, there was a correlation between breast volume (cutoff 700 cc) and acute toxicity, however this was not the case for HF-SIB. Unfortunately, information on breast volume was not available in our dataset. Chronic skin toxicity was overall favorable and also lower with HF-SIB. Long-term cosmetic outcome was good or excellent in > 90% of patients in both groups.

The second study by Lertbutsayanukul et al. [31] analyzed 114 patients treated with NF-SIB (50/65 Gy in 25 fractions) or HF-SIB (43.2 Gy and 52.8 Gy in 16 fractions), both of which were applied with 3D-CRT. Their findings concur with Fiorentino et al. and demonstrate reduced acute toxicity with HF-SIB. Grade 1/2 radiation dermatitis was 91.3% with NF-SIB and 73.7% with HF-SIB.

Recently, Borm and colleagues have shown that dosimetric parameters might predict the development of radiation dermatitis for 3D-CRT [32], however these findings were not yet validated for IMRT.

There are several limitations regarding the results of this analysis, the most important being the retrospective data collection and the short-term follow-up. However, patients were treated on a prospective protocol with relatively homogenous treatment planning and clinical assessment at one large tertiary hospital. A relevant number of patients on Arm B received a lower boost dose. This might have biased the results in favor of the standard arm and might explain the lower incidence of radiation dermatitis in Arm B. However, multi-angle IMRT-techniques can lead to increased skin surface doses due to a reduced build-up effect [33]. Pain is a subjective outcome parameter. The lack of assessment with a quantification tool such as the visual analogue scale might impair the reproducibility and prevalence. A detailed analysis of the prospectively collected quality of life-questionnaires within this trial is planned. Lymphedema is a frequent complication of breast cancer treatment, especially in patients who received axillary dissection and chemotherapy [34]. The score used in this analysis has not been validated, however it represents a clinically relevant measure since it incorporates both symptoms and treatment for lymphedema.

At the time of conception of the IMRT-MC2-trial, normofractionated radiotherapy was the standard of care after breast-conserving surgery for breast cancer. During the conduct of the trial, hypofractionated radiotherapy was shown to be equally effective in terms of tumor control with a slightly lower risk of acute and chronic cutaneous and subcutaneous side effects [35], a reduction in treatment time and better health economics [36]. However, for several subgroups, normofractionated radiotherapy is still regarded as the standard of care, especially patients who are planned to receive regional nodal irradiation. Some authors also question the use of hypofractionated radiotherapy for patients who receive HER2-targeted therapy, young patients, patients with very large breasts, patients who underwent neoadjuvant chemotherapy [37] and patients with connective tissue disorders [38]. Despite accumulating data for hypofractionated post-mastectomy radiotherapy [39], normofractionation is still considered the standard of care in this setting. Thus, it is prudent to argue that normofractionated radiotherapy will still have a place in adjuvant radiotherapy for breast cancer in the foreseeable future. Several trials regarding HF-SIB are ongoing [40].

## Conclusion

Both 3D-CRT-seqB and IMRT-SIB with normofractionated whole-breast radiotherapy were well tolerated in the adjuvant treatment after BCS, however the differences in terms of radiodermatitis and breast pain merit further consideration. Analyses regarding late toxicity, quality of life and recurrence rates are ongoing and are crucial to determine the future role of IMRT-SIB.

## Data Availability

The datasets analysed during the current study are available from the corresponding author on reasonable request.

## Abbreviations

3D-CRT: Three dimensional-conformal radiotherapy
BCS: Breast-conserving surgery
CONSORT: Consolidated Standards of Reporting Trials
CTCAE: Common Terminology Criteria for Adverse Events
EORTC: European Organization for the Research and Treatment of Cancer
IMRT: Intensity-modulated radiotherapy
LENT-SOMA: Late effects of Normal Tissue - Subjective Objective Management Analysis
seqB: Sequential boost
SIB: Simultaneous integrated boost

## References

[1] Darby S, McGale P, Correa C, Taylor C, Arriagada R, Early Breast Cancer Trialists' Collaborative Group (EBCTCG) (2011). Effect of radiotherapy after breast-conserving surgery on 10-year recurrence and 15-year breast cancer death: meta-analysis of individual patient data for 10,801 women in 17 randomised trials. Lancet.

[2] Bartelink H, Maingon P, Poortmans P, Weltens C, Fourquet A, Jager J (2015). Whole-breast irradiation with or without a boost for patients treated with breast-conserving surgery for early breast cancer: 20-year follow-up of a randomised phase 3 trial. Lancet Oncol.

[3] Haviland JS, Owen JR, Dewar JA, Agrawal RK, Barrett J, Barrett-Lee PJ (2013). The UK Standardisation of Breast Radiotherapy (START) trials of radiotherapy hypofractionation for treatment of early breast cancer: 10-year follow-up results of two randomised controlled trials. Lancet Oncol.

[4] Owen JR, Ashton A, Bliss JM, Homewood J, Harper C, Hanson J (2006). Effect of radiotherapy fraction size on tumour control in patients with early-stage breast cancer after local tumour excision: long-term results of a randomised trial. Lancet Oncol.

[5] Shaitelman SF, Lei X, Thompson A, Schlembach P, Bloom ES, Arzu IY, et al. Three-Year Outcomes With Hypofractionated Versus Conventionally Fractionated Whole-Breast Irradiation: Results of a Randomized, Noninferiority Clinical Trial. J Clin Oncol. 2018;36(35):3495–503. 10.1200/JCO.18.00317 PMC6286164 30379626

[6] Aly MMOM, Glatting G, Jahnke L, Wenz F, Abo-Madyan Y (2015). Comparison of breast simultaneous integrated boost (SIB) radiotherapy techniques. Radiat Oncol.

[7] Hurkmans CW, Meijer GJ, van Vliet-Vroegindeweij C, van der Sangen MJ, Cassee J (2006). High-dose simultaneously integrated breast boost using intensity-modulated radiotherapy and inverse optimization. Int J Radiat Oncol Biol Phys.

[8] Van Parijs H, Reynders T, Heuninckx K, Verellen D, Storme G, De Ridder M (2014). Breast conserving treatment for breast cancer: dosimetric comparison of different non-invasive techniques for additional boost delivery. Radiat Oncol.

[9] Van Parijs H, Reynders T, Heuninckx K, Verellen D, Storme G, De Ridder M (2014). Breast conserving treatment for breast Cancer: Dosimetric comparison of sequential versus simultaneous integrated photon boost. Biomed Res Int.

[10] Donovan E, Bleakley N, Denholm E, Evans P, Gothard L, Hanson J (2007). Randomised trial of standard 2D radiotherapy (RT) versus intensity modulated radiotherapy (IMRT) in patients prescribed breast radiotherapy. Radiother Oncol.

[11] Pignol J-P, Olivotto I, Rakovitch E, Gardner S, Sixel K, Beckham W (2008). A multicenter randomized trial of breast intensity-modulated radiation therapy to reduce acute radiation dermatitis. J Clin Oncol.

[12] Pignol J-P, Truong P, Rakovitch E, Sattler MG, Whelan TJ, Olivotto IA (2016). Ten years results of the Canadian breast intensity modulated radiation therapy (IMRT) randomized controlled trial. Radiother Oncol.

[13] Barnett GC, Wilkinson J, Moody AM, Wilson CB, Sharma R, Klager S (2009). A randomised controlled trial of forward-planned radiotherapy (IMRT) for early breast cancer: baseline characteristics and dosimetry results. Radiother Oncol.

[14] Mukesh MB, Barnett GC, Wilkinson JS, Moody AM, Wilson C, Dorling L (2013). Randomized controlled trial of intensity-modulated radiotherapy for early breast Cancer: 5-year results confirm superior overall Cosmesis. J Clin Oncol.

[15] Askoxylakis V, Jensen AD, Häfner MF, Fetzner L, Sterzing F, Heil J (2011). Simultaneous integrated boost for adjuvant treatment of breast cancer- intensity modulated vs. conventional radiotherapy: the IMRT-MC2 trial. BMC Cancer.

[16] Pezner RD, Patterson MP, Hill LR, Vora N, Desai KR, Archambeau JO (1985). Breast retraction assessment: an objective evaluation of cosmetic results of patients treated conservatively for breast cancer. Int J Radiat Oncol Biol Phys.

[17] Vrieling C, Collette L, Bartelink E, Borger JH, Brenninkmeyer SJ, Horiot JC (1999). Validation of the methods of cosmetic assessment after breast-conserving therapy in the EORTC “boost versus no boost” trial. EORTC Radiotherapy and Breast Cancer Cooperative Groups European Organization for Research and Treatment of Cancer. Int J Radiat Oncol Biol Phys.

[18] Harris JR, Levene MB, Svensson G, Hellman S (1979). Analysis of cosmetic results following primary radiation therapy for stages I and II carcinoma of the breast. Int J Radiat Oncol Biol Phys.

[19] Mak KS, Chen Y-H, Catalano PJ, Punglia RS, Wong JS, Truong L (2015). Dosimetric inhomogeneity predicts for long-term breast pain after breast-conserving therapy. Int J Radiat Oncol Biol Phys.

[20] Bantema-Joppe EJ, Schilstra C, de Bock GH, Dolsma WV, Busz DM, Langendijk JA (2012). Simultaneous integrated boost irradiation after breast-conserving surgery: physician-rated toxicity and cosmetic outcome at 30 months' follow-up. Int J Radiat Oncol Biol Phys.

[21] Bantema-Joppe EJ, Vredeveld EJ, de Bock GH, Busz DM, Woltman-van Iersel M, Dolsma WV (2013). Five year outcomes of hypofractionated simultaneous integrated boost irradiation in breast conserving therapy; patterns of recurrence. Radiother Oncol.

[22] McDonald MW, Godette KD, Whitaker DJ, Davis LW, Johnstone PAS (2010). Three-year outcomes of breast intensity-modulated radiation therapy with simultaneous integrated boost. Int J Radiat Oncol Biol Phys.

[23] Pasquier D, Le Tinier F, Bennadji R, Jouin A, Horn S, Escande A (2019). Intensity-modulated radiation therapy with simultaneous integrated boost for locally advanced breast cancer: a prospective study on toxicity and quality of life. Sci Rep.

[24] Meng J, Huang W, Mei X, Yu X, Pan Z, Ma J (2020). Adjuvant breast inversely planned intensity-modulated radiotherapy with simultaneous integrated boost for early stage breast cancer : Results from a phase II trial. Strahlenther Onkol.

[25] Vasiljevic D, Arnold C, Neuman D, Fink K, Popovscaia M, Kvitsaridze I (2018). Occurrence of pneumonitis following radiotherapy of breast cancer - a prospective study. Strahlenther Onkol.

[26] Lee H-H, Hou M-F, Chuang H-Y, Huang M-Y, Tsuei L-P, Chen F-M (2015). Intensity modulated radiotherapy with simultaneous integrated boost vs. conventional radiotherapy with sequential boost for breast cancer - a preliminary result. Breast.

[27] Paelinck L, Gulyban A, Lakosi F, Vercauteren T, De Gersem W, Speleers B (2017). Does an integrated boost increase acute toxicity in prone hypofractionated breast irradiation? A randomized controlled trial. Radiother Oncol.

[28] Van Parijs H, Miedema G, Vinh-Hung V, Verbanck S, Adriaenssens N, Kerkhove D (2012). Short course radiotherapy with simultaneous integrated boost for stage I-II breast cancer, early toxicities of a randomized clinical trial. Radiat Oncol.

[29] Shaitelman SF, Schlembach PJ, Arzu I, Ballo M, Bloom ES, Buchholz D (2015). Acute and short-term toxic effects of conventionally fractionated vs Hypofractionated whole-breast irradiation. JAMA Oncol.

[30] Fiorentino A, Gregucci F, Mazzola R, Figlia V, Ricchetti F, Sicignano G (2018). Intensity-modulated radiotherapy and hypofractionated volumetric modulated arc therapy for elderly patients with breast cancer: comparison of acute and late toxicities. Radiol Med.

[31] Lertbutsayanukul C, Pitak M, Ajchariyasongkram N, Rakkiet N, Seuree F, Prayongrat A (2020). Long-term patient-rated cosmetic and satisfactory outcomes of early breast cancer treated with conventional versus hypofractionated breast irradiation with simultaneous integrated boost technique. Breast J.

[32] Borm KJ, Loos M, Oechsner M, Mayinger MC, Paepke D, Kiechle MB, et al. Acute radiodermatitis in modern adjuvant 3D conformal radiotherapy for breast cancer - the impact of dose distribution and patient related factors. Radiat Oncol. 2018:1–7. 10.1186/s13014-018-1160-5 PMC6223003 30404664

[33] Higgins PD, Han EY, Yuan JL, Hui S, Lee CK (2007). Evaluation of surface and superficial dose for head and neck treatments using conventional or intensity-modulated techniques. Phys Med Biol.

[34] Rupp J, Hadamitzky C, Henkenberens C, Christiansen H, Steinmann D, Bruns F (2019). Frequency and risk factors for arm lymphedema after multimodal breast-conserving treatment of nodal positive breast Cancer - a long-term observation. Radiat Oncol.

[35] Hickey BE, James ML, Lehman M, Hider PN, Jeffery M, Francis DP (2016). Fraction size in radiation therapy for breast conservation in early breast cancer. Cochrane Database Syst Rev.

[36] Deshmukh AA, Shirvani SM, Lal L, Swint JM, Cantor SB, Smith BD (2017). Cost-effectiveness analysis comparing conventional, Hypofractionated, and intraoperative radiotherapy for early-stage breast Cancer. J Natl Cancer Inst.

[37] Krug D, Baumann R, Budach W, Dunst J, Feyer P, Fietkau R (2018). Individualization of post-mastectomy radiotherapy and regional nodal irradiation based on treatment response after neoadjuvant chemotherapy for breast cancer : a systematic review. Strahlenther Onkol.

[38] Recht A, McArthur H, Solin LJ, Tendulkar R, Whitley A, Giuliano A (2019). Contemporary guidelines in whole-breast irradiation: an alternative perspective. Int J Radiat Oncol Biol Phys.

[39] Liu L, Yang Y, Guo Q, Ren B, Peng Q, Zou L (2020). Comparing hypofractionated to conventional fractionated radiotherapy in postmastectomy breast cancer: a meta-analysis and systematic review. Radiat Oncol.

[40] Nitsche M, Dunst J, Carl UM, Hermann RM (2015). Emerging role of Hypofractionated radiotherapy with simultaneous integrated boost in modern radiotherapy of breast Cancer. Breast Care.

